# Investigating the importance of individual mitochondrial genotype in susceptibility to drug-induced toxicity

**DOI:** 10.1042/BST20190233

**Published:** 2020-05-26

**Authors:** Sophie L. Penman, Alice S. Carter, Amy E. Chadwick

**Affiliations:** MRC Centre for Drug Safety Science, Department of Molecular and Clinical Pharmacology, University of Liverpool, Liverpool L69 3GE, U.K.

**Keywords:** adverse drug reactions, antiretroviral, haplogroup, mitochondrial dysfunction, mtDNA

## Abstract

The mitochondrion is an essential organelle responsible for generating cellular energy. Additionally, mitochondria are a source of inter-individual variation as they contain their own genome. Evidence has revealed that mitochondrial DNA (mtDNA) variation can confer differences in mitochondrial function and importantly, these differences may be a factor underlying the idiosyncrasies associated with unpredictable drug-induced toxicities. Thus far, preclinical and clinical data are limited but have revealed evidence in support of an association between mitochondrial haplogroup and susceptibility to specific adverse drug reactions. In particular, clinical studies have reported associations between mitochondrial haplogroup and antiretroviral therapy, chemotherapy and antibiotic-induced toxicity, although study limitations and conflicting findings mean that the importance of mtDNA variation to toxicity remains unclear. Several studies have used transmitochondrial cybrid cells as personalised models with which to study the impact of mitochondrial genetic variation. Cybrids allow the effects of mtDNA to be assessed against a stable nuclear background and thus the *in vitro* elucidation of the fundamental mechanistic basis of such differences. Overall, the current evidence supports the tenet that mitochondrial genetics represent an exciting area within the field of personalised medicine and drug toxicity. However, further research effort is required to confirm its importance. In particular, efforts should focus upon translational research to connect preclinical and clinical data that can inform whether mitochondrial genetics can be useful to identify at risk individuals or inform risk assessment during drug development.

## Introduction

The mitochondrion is an important organelle in the evolution of eukaryotic cells. The main function of which is the generation of cellular energy in the form of adenosine triphosphate (ATP) via two coupled processes, the oxidation of reduced electron carriers and the phosphorylation of adenosine diphosphate (ADP). Mitochondria are also involved in other pivotal cellular processes including the regulation of apoptosis, the production of reactive oxygen species, steroid and heme synthesis and calcium (Ca^2+^) signalling [1,2]. Furthermore, mitochondria are a source of inter-individual variation as they contain their own genome. The mitochondrial genome encodes core subunits of the electron transport chain (ETC) and it has been shown that variation in mtDNA can give rise to differences in mitochondrial function [3–5].

The detection of adverse drug reactions (ADRs) post-market is a major burden to the clinic and the pharmaceutical industry due to the high economic cost of drug-development and the severe life-threatening conditions that may manifest [6–8]. Idiosyncratic drug-induced toxicities pose the most uncertainty to drug development as they do not occur in most patients treated with the drug and have no clear dose-dependent relationship [9]. Idiosyncratic ADRs typically only occur when the novel therapeutic is tested on a large population post-market.

The mitochondria possess a wealth of structural moieties and functional features which can be targeted by a compound and lead to toxicity [10]. These include ETC inhibition, uncoupling of oxidative phosphorylation, the opening of the mitochondrial permeability transition pore, alterations in mitochondrial dynamics and the depletion of the mitochondrial genome [10,11]. A vast amount of research suggests that drug-induced mitochondrial dysfunction plays an important role in a variety of drug-induced organ toxicities [11,12]. Due to the role of mitochondrial dysfunction in the onset of ADRs, it has been hypothesised that variation in mtDNA may underpin some of the idiosyncrasies associated with drug-induced toxicity by offering another source of inter-individual variation [10]. This article will review the current literature investigating this topic and consider how future research could advance the understanding of this field, in particular its translational importance to improving medicine safety.

### An overview of mitochondrial genetics

**Figure 1.**
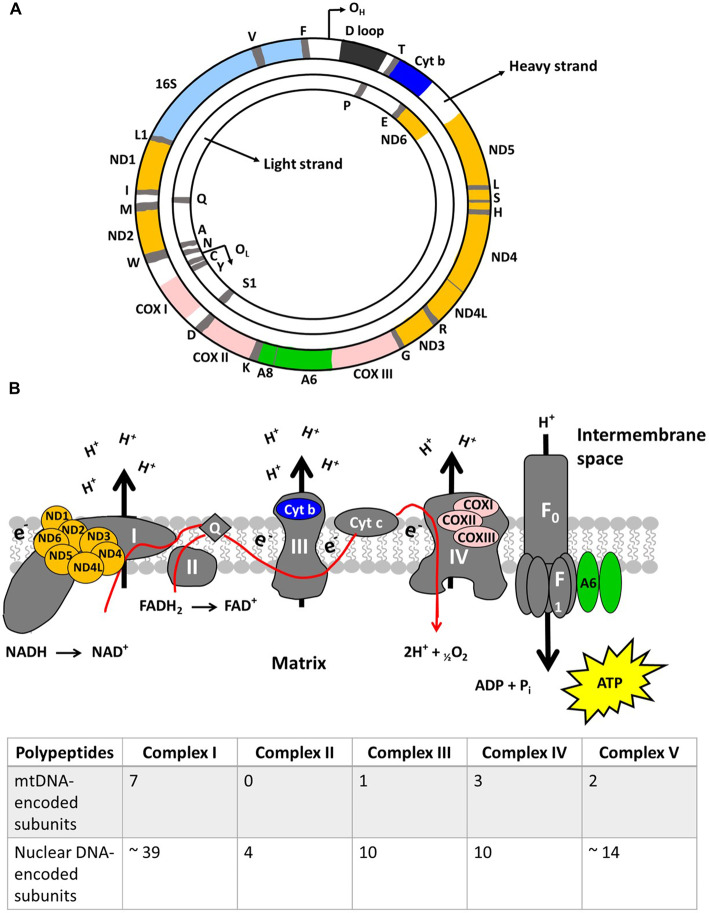
Mitochondrial DNA (mtDNA) structure and schematic representation of the electron transport chain and the proteins encoded by the mtDNA. (A) Human mtDNA is double-strand, circular molecule responsible for encoding 37 genes. (B) The mtDNA encodes 13 proteins, which are shown in colour. The colours correspond to the colours of the polypeptide-coding gene in Figure 1A. Abbreviations: ND, NADH dehydrogenase; Q, ubiquinone; cyt b, cytochrome *b*; COX, cytochrome *c* oxidase; A, ATPase; O_H_, origins of replication of the heavy strand; O_L_, origins of replication of the light strand. Adapted from [16].

MtDNA is a double-stranded, circular molecule composed of 16 569 base pairs [13]. MtDNA contains only 37 genes of which 13 encode for proteins, 22 encode for transfer RNA (tRNA) and two ribosomal RNA (rRNA) [14]. The 13 proteins encoded for by mtDNA are all components of the ETC, with the remaining respiratory chain subunits being encoded for by the nuclear genome (Figure 1) [13]. MtDNA is packaged into DNA–protein complexes termed mitochondrial nucleoids and on average, each nucleoid contains 1.4 mtDNA molecules [15]. Each mitochondrion contains thousands of copies of mtDNA but the exact numbers vary in accordance with the bioenergetic needs of the tissue [16].

MtDNA is known to have a high mutation rate and due to a process called heteroplasmy, it is common for cells to contain both wild-type mtDNA and mutant mtDNA [17]. Mutations in mtDNA can accumulate over the lifetime of an individual but are unlikely to instigate a cellular phenotype until a critical threshold is reached, estimated to be 60–80% [16,17].

MtDNA is primarily maternally inherited and the progressive accumulation of mtDNA single nucleotide polymorphisms (SNPs) through the maternal lineage has led to the creation of mitochondrial haplogroups which are characterised by groups of individuals sharing groups of mitochondrial genes with similar patterns of SNPs which were inherited from our ancestral ‘mitochondrial Eve’ [17–19]. The maternal inheritance of mtDNA, combined with the evolutionary spread of humans across the globe and the lack of recombination, has led to specific mitochondrial haplogroups being associated with different ethnic groups [17,20]. Advances in the ability to study the mitochondrial genome has led to the development of the phylogenetic tree, which has identified more division within the macro-haplogroups termed sub-haplogroups or subclades [17,19].

## Mitochondrial genetics in the context of drug-induced toxicity

Within the last 20 years, knowledge and understanding of the mitochondrial genome has greatly expanded [21]. In particular, much research has focused upon the link between mitochondrial genetics and the manifestation of disease. The mitochondrial genome encodes core subunits of the ETC and thus, variation within mtDNA can affect mitochondrial bioenergetics [3–5]. In the context of disease, mtDNA mutations can result in a change in cellular energy metabolism, resulting in a pathogenic phenotype [21]. For example, associations have been observed between mitochondrial haplogroups and predisposition to Parkinson's disease, multiple sclerosis, Leber hereditary optic neuropathy, type 1 and type 2 diabetes, cardiomyopathies and stroke-like episodes [22,23]. Additionally, multiple studies have revealed that there is an association between improved CD4+ count following antiretroviral therapy (ART) and mtDNA haplogroups revealing that mitochondrial genetics are also implicated in differential drug efficacy [24–28]. Given the association of mitochondrial haplogroups, disease and drug efficacy, research has progressed to elucidate the role of mtDNA variation in susceptibility to drug toxicity. Differences in mitochondrial function due to mtDNA variation has the potential to lead to idiosyncratic drug-induced toxicity [10]. Studies elucidating the effects of mitochondrial haplogroups on susceptibility to drug-induced toxicity are limited but the most well studied therapeutic classes are ART, antibiotics and chemotherapeutic agents, which will, therefore, be the focus for this review.

### Antiretroviral therapy

Human immunodeficiency virus (HIV) is a chronic blood-borne retrovirus that infects host CD4+ T-cells, resulting in their depletion and immunosuppression. If HIV is not treated, individuals can progress to acquired immune deficiency syndrome (AIDS) [29]. Progression of HIV to AIDS and the overall mortality rate has been greatly reduced through the early use of ART as part of the highly active antiretroviral therapy (HAART) regimen [30]. However, 25% of patients discontinue HAART due to resistance, non-compliance and toxicity [31]. ARTs are associated with a variety of ADRs including lipoatrophy, peripheral neuropathy, cardiomyopathy, lactic acidosis and metabolic abnormalities and mechanistic studies have revealed that many ARTs induce toxicity via an effect upon the mitochondria [27,32,33]. Therefore, research has begun to investigate whether mtDNA, in particular at the haplogroup level, is a factor underlying susceptibility to ART-induced toxicity, summarised in Table 1.

**Table 1.** Mitochondrial haplogroups or mtDNA variants associated with ART-induced toxicity

Reference	Haplogroup/mtDNA variants associated with ART toxicity	Description of studies and findings
Peripheral neuropathy		
[33]	T	Retrospective case-control study involving 509 participants all belonging to European haplogroups. 17.1% of individuals who developed peripheral neuropathy belonged to haplogroup T. The risk of toxicity was associated with ddI and d4T.
[34]	L1c	A race-specific, case-control study involving 156 non-Hispanic black participants. 33% of participants developed peripheral neuropathy with haplogroup L1c and ddI and d4T treatment increasing the likelihood of toxicity.
Metabolic complications		
[38]	U, T and JT	A study of 248 patients co-infected with HIV and hepatitis C virus from Spain. Patients belonging to haplogroup U were an increased risk of insulin resistance and haplogroup JT or T had an increased risk of atherogenic dyslipidaemia.
[36]	I	A retrospective study of 231 white, non-Hispanic participants on efavirenz or lopinavir. Haplogroup I was associated with an increase in lipid levels and likelihood to develop lipoatrophy.
[37]	mtDNA variant m.10398A>G	A pilot study of 30 white, non-Hispanic participant. Participants with the mtDNA variant m.10398A>G were associated with a lower FMD implying impaired endothelial function and decreased levels of adiponectin, suggestive of potential cardiovascular toxicity.
[32]	H	A multicentre study of 410 Caucasian participants. Haplogroup H was associated with an increased risk of lipoatrophy in the arms, legs and buttocks. Participants belonging to haplogroup T were borderline protective against lipoatrophy.
[39]	None	A retrospective study of 346 HIV positive patients with ART related lipodystrophy. There were no significant associations between measures of lipodystrophy and mtDNA haplogroups.

Sensory neuropathy is the most common neurological complication associated with ART [33]. A retrospective case-control study analysing the data of 509 Caucasian individuals with European haplogroups from the Adults AIDS clinical trials group (ACTG) 384 concluded that participants belonging to haplogroup T were associated with a higher susceptibility to peripheral neuropathy following treatment with didanosine (ddI) and stavudine (d4T) [33]. In a subsequent study, using the same ACTG 384 cohort of patients but with the addition of 156 individuals who self-identified as non-Hispanic black (all three major African haplogroups L1, L2 and L3 were present), it was concluded that participants belonging to haplogroup L1 were associated with a greater risk of developing peripheral neuropathy from ddI and d4T [34]. However, a prospective observational cohort study of 549 participants from the US-based ‘CNS HIV Antiretroviral Therapy Effects Research’ (CHARTER) reported contradictory results; participants belonging to haplogroup J and L1 were associated with a decreased occurrence of peripheral neuropathy compared with other haplogroups [35]. A potential caveat of both of these studies is that susceptibility was considered in comparison with other haplogroups, rather than as a risk in isolation, and consequently results are dependent upon the selected study population. This highlights the need for additional studies with greater sample sizes and haplogroup variation in order to confirm the susceptibility of peripheral neuropathy for haplogroup L1. Furthermore, ddI and d4T are no longer recommended first-line treatment and so research based upon the currently recommended treatment regimens should be prioritised.

Studies assessing the association between metabolic disturbances and haplogroup have been conducted in small Caucasian populations and have revealed differing risks of toxicity amongst haplogroups and for specific mtDNA variants [36–38]. A cross-sectional study of 248 patients from Spain, co-infected with HIV and hepatitis C virus, revealed an association between European haplogroups and the risk of insulin resistance and atherogenic dyslipidaemia [38]. In particular, patients belonging to haplogroup U had an increased risk of insulin resistance, whilst patients belonging to haplogroup JT or T had an increased risk of atherogenic dyslipidaemia following HAART treatment [38]. A retrospective study of 231 white, non-Hispanic participants from a study examining the efficacy of either efavirenz or lopinavir–ritonavir in HAART treatment revealed that mitochondrial haplogroups were associated with ART-induced changes in serum lipids and body composition in HIV patients [36]. Participants belonging to haplogroup I were associated with the greatest changes in lipid levels and were more likely to develop lipoatrophy than other non-I haplogroups [36]. In a multicentre study of 410 self-identified, white HIV positive participants on HAART, it was revealed that individuals belonging to haplogroup H were associated with an increase in lipoatrophy in the arms, buttocks and legs whereas those belonging to haplogroup T were borderline protected against lipoatrophy [32]. Conversely, in a retrospective study of 346 white HIV positive participants, it was concluded that there were no significant associations between ART-induced lipodystrophy and haplogroups [39]. Following on from this, a pilot study explored associations between mitochondrial haplogroup and changes in endothelial function [37]. White, non-Hispanic participants (30 participants) were recruited and temporal changes in brachial artery flow-mediated dilation (FMD) and cardiovascular biomarkers, including adiponectin were measured. Whilst no haplogroup demonstrated an increased risk of developing toxicity, individuals with the mtDNA variant m.10398A>G were at an increased risk of short-term metabolic dysfunction due to ART toxicity. Subjects with the mtDNA polymorphism m.10398A>G were found to have a lower FMD at week 24 of the study, indicating impaired endothelial function. Additionally, m.10398A>G was associated with significantly decreased levels of adiponectin at week 24, suggestive of potential cardiovascular toxicity. Notably, the m.10398A>G variant is present in the European haplogroups I, J and K. Given the association between m.10398A>G and short-term metabolic dysfunction, the need for larger-scale research to be undertaken to elucidate the association between m.10398A>G and long-term ART-induced toxicity is suggested.

### Chemotherapeutic agents

Chemotherapeutic agents are employed in the treatment and management of many cancers; however, the majority of patients experience some form of ADR [40]. Interestingly, disease pathophysiology has been related to the mitochondrial genome; SNPs in the mitochondrial genome have been revealed to enhance susceptibility to sporadic breast cancer in various populations [41]. Additionally, specific mitochondrial haplogroups or the presence of mutations in germline mtDNA can increase the prevalence of oesophageal, breast and pancreatic cancer [42,43]. Given the association between mtDNA variation and cancer susceptibility, the hypothesis that mitochondrial genetics may also confer differences in the onset of chemotherapeutic toxicity is also under investigation [43].

Cisplatin is a commonly used platinum-based alkylating chemotherapy agent that acts through DNA synthesis interference and the induction of cellular apoptosis [44]. Cisplatin is used to treat a broad range of tumours including ovarian, testicular, lung, cervical, bladder and head and neck [45]. Despite its efficacy, cisplatin is associated with ADRs including nephrotoxicity, ototoxicity and retinopathy [46]. Thus far, one pre-clinical study and two clinical studies have assessed the effects of mtDNA variation on cisplatin-induced efficacy and toxicity [46–48].

**Figure 2.**
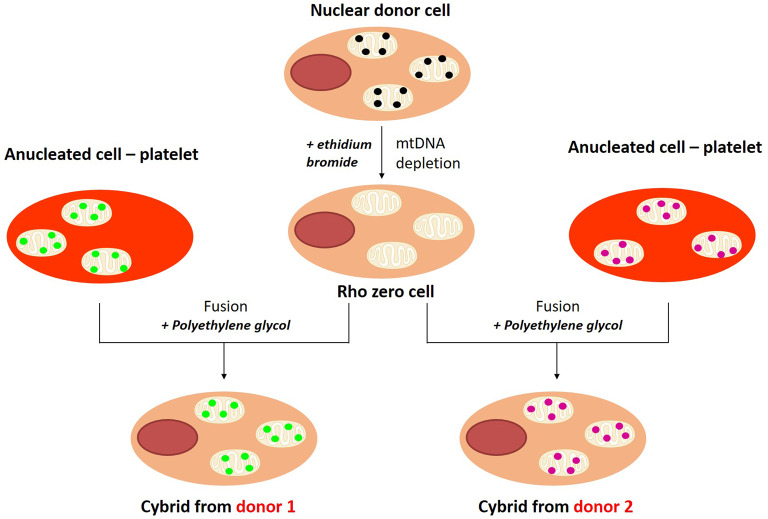
The generation of transmitochondrial cybrids from ρ0 cells and platelets. A nuclear donor cell is devoid of its mtDNA using the DNA-intercalator ethidium bromide to produce a ρ0 cell. Platelets are anucleated cells and can be isolated from patient blood donations. Platelets are fused with ρ0 cells using polyethylene glycol to produce transmitochondrial cells. Both cybrids will have the same nuclear DNA background but will have different mtDNA from the different platelet donors.

Transmitochondrial cybrids were used as an *in vitro* model to study the role of individual mitochondrial genotype in cisplatin-induced retinopathy [46]. Transmitochondrial cybrids are unique as their generation enables the impact of mitochondrial genotype to be assessed against a constant nuclear background (Figure 2) [49]. In their study, Patel et al. generated retinal epithelial (RPE) transmitochondrial cybrids specific for haplogroup H and J [46]. At baseline, RPE cybrids belonging to haplogroup J exhibited a faster growth rate than haplogroup H RPE cybrids. Haplogroup J-RPE cybrids were associated with a greater sensitivity to cisplatin as they experienced the greatest decline in viability and mitochondrial membrane potential compared with H-cybrids. Additionally, RPE cybrids belonging to haplogroup J demonstrated an up-regulation of genes involved in apoptosis following cisplatin treatment, including BCL2-associated X protein (BAX) and caspase-3 compared with H-cybrids. Conversely, H-cybrids demonstrated a significant elevation in cyclin-dependent kinase inhibitor 1A following cisplatin treatment which is implicated in drug toxicity. The observation of haplogroup-specific responses to cisplatin treatment offers preliminary data supporting the role of mtDNA as a factor driving mechanistic differences in the cellular response to cisplatin and that transmitochondrial cybrids offer a useful model to define the mechanisms underlying genotype-specific differences in susceptibility to compound toxicity.

Cisplatin-induced ototoxicity is thought to arise from the off-target formation of cisplatin adducts with mtDNA. Two clinical studies have been conducted to investigate links between mtDNA variation and ototoxicity, although with contrasting results [47,48]. Graterol et al. concluded that there were no significant associations between haplogroup and ototoxicity in an observational cohort study of 72 Spanish Caucasian cancer patients [47]. Conversely, Peters et al. sequenced the mtDNA of 39 patients on cisplatin therapy and concluded that hearing impairment secondary to cisplatin treatment was associated with haplogroup J [48]. However, both of these observations are based upon small sample sizes and therefore larger studies may be required to investigate the link further.

### Antibiotics

Aminoglycosides are broad-spectrum antibiotics which function to inhibit bacterial ribosomes thus leading to bacterial protein synthesis suppression [50]. Whilst a valuable class of antibiotics, aminoglycosides induce ADRs in many patients. Ototoxicity and nephrotoxicity are common ADRs associated with aminoglycosides and mitochondrial dysfunction has been demonstrated as an off-target adverse effect of aminoglycosides [51,52]. It is hypothesised that the bacterial evolution of mitochondria has led to a close resemblance between bacterial and mitochondrial ribosomes thus resulting in an unintended interruption of mitochondrial ribosomal function in some patients [53].

Individuals harbouring the mutation m.1555A>G in the mitochondrial genome are predisposed to aminoglycoside-induced ototoxicity [54]. The m.1555A>G mutation occurs in the gene encoding the 12s rRNA and results in the mitochondrial ribosome being more structurally similar to the bacterial ribosome [53]. The prevalence of the m.1555A>G mutation was examined in two Japanese groups where it was concluded that 33–59% of all aminoglycoside-related toxicities were due to the m.1555A>G mutation [55]. Additional evidence has shown that there is also an association between the m.1555A>G mutation and aminoglycoside-induced ototoxicity in the Spanish and Cuban population [56,57]. Specifically, in the study performed in the Spanish population in individuals with the m.1555A>G mutation, the probability of developing deafness by the age of 30 was 96.5% if the individual had received an aminoglycoside compared with 39.9% if they have never received treatment [56]. An additional mutation located in the mitochondrial genome and associated with aminoglycoside-induced toxicity is the m.1494C>T mutation. The m.1494C>T mutation in the decoding site of the 12S rRNA can also enhance the similarity of the mitochondrial ribosome to that of bacterial ribosomes [53]. Zhao et al. generated osteosarcoma transmitochondrial cybrids from a Chinese family with a history of aminoglycoside-induced ototoxicity, which revealed that the m.1494C>T mutation was the primary factor responsible for impairments in mitochondrial protein synthesis [58,59]. Furthermore, these cybrids when treated with the aminoglycoside paromomycin also had an enhanced toxicity compared with cybrids without the m.1494C>T mutation as evidenced by a reduced growth rate [58]. Despite evidence of the m.1555A>G and m.1494C>T mutations playing significant roles in aminoglycoside-induced ototoxicity, the presence of these mutations alone is insufficient to produce the clinical phenotype of deafness as evidenced by a varied penetrance pattern [53,60]. It has been concluded that nuclear-modifier genes may alter the phenotypic manifestation of both the m.1555A>G and m.1494C>T mutation by either up-regulation or suppression of the effects of the mutation [60]. However, these nuclear genes are not directly linked to aminoglycoside-induced ototoxicity and so there is a need for further research to advance our understandings of the complex link between nuclear-modifier genes and mtDNA mutations. Given the strong evidence for the association between mtDNA mutations and aminoglycoside-induced ototoxicity, there could be potential for the stratification of treatment. Within the United Kingdom, it has been estimated that the cost of testing for the m.1555A>G mutation would be £35 [54]. This cost is significantly less than the cost of rehabilitation of deaf individuals and it has been postulated that its deployment could prevent reductions in quality of life resulting from ototoxicity [54].

Linezolid belongs to the antibiotic class oxazolidinones and is commonly used in the treatment of respiratory tract and skin infections [61]. Linezolid inhibits bacterial protein synthesis by binding to the 23S rRNA of prokaryotic ribosomes and thus it is hypothesised that toxicity secondary to linezolid can occur due to an interference with mitochondrial ribosomes, similarly to aminoglycosides [62]. Whilst safe for short durations, long-term therapy is associated with ADRs such as myelosuppression, optic atrophy, peripheral neuropathy and metabolic disturbances [62]. The association between mtDNA variation and linezolid toxicity has been researched in a clinical setting. Garrabou et al. conducted an observational and longitudinal follow-up of 19 Spanish patients before and after linezolid treatment for 28 days in which linezolid toxicity was determined based upon clinical symptoms of toxicity or reduced mitochondrial protein synthesis [62]. It was reported that patients belonging to haplogroup H had a reduced incidence of toxicity whilst haplogroup U was associated with a greater risk of toxicity [62]. However, in the patient cohort, only haplogroups H and U were statistically represented and whilst participants belonging to other haplogroups were present, they were scarcely represented amongst a limited, small sample size.

*In vitro* studies to assess the role of mitochondrial genotype in susceptibility to linezolid toxicity were performed using 25-separate osteosarcoma transmitochondrial cybrids generated from common European haplogroups (H (non H1), H1, J1, Uk and T) [63]. Cybrids generated for haplogroup J1 were more susceptible to linezolid-induced toxicity. Furthermore, two polymorphisms, m.2706A>G and m.3010G>A, located within regions encoding for 16S rRNA, were confirmed to modify susceptibility to linezolid. Importantly, these alleles are close to the ribosomal peptidyl transferase centre, which is where several antibiotics bind [64]. The m.3010G>A polymorphism is not haplogroup specific and can be found within haplogroups H1 and J1 in the European population, haplogroup D4 in the Chinese, Asian and Native American population and haplogroup L2a1c in the African population [63,65,66]. Given the apparent worldwide risk population containing these susceptible mtDNA variants, there is a need for further research to advance understandings of the exact association between these mtDNA variants and the likelihood of clinical ADRs.

## Conclusions and future directions

Current evidence supports that there is a link between mtDNA variation and some forms of drug-induced ADRs. However, so far, its importance to the clinical setting remains unclear due to limitations in study design, particularly the size and genotypic diversity. As a field, mitochondrial genetics is rapidly growing, fuelled by advancements in mitochondrial genotyping technology and analysis, leading to a much more in-depth understanding of mitochondrial genetics. For example, in the past, it was common to conduct targeted sequencing of the mitochondrial genome using primers for pre-chosen SNPs that are characteristic of a haplogroup. However, next-generation sequencing (NGS) is now a commonly used technique, which provides the whole mitochondrial genome sequence enabling sub-haplogroups to be identified as well as providing information on levels of heteroplasmy and mitochondrial variants [23,67,68]. Following NGS, mtDNA sequences are compared with reference sequences in a process called variant calling in order to identify true variants from ‘noise’ [69]. As previously mentioned, each cell can contain hundreds to thousands of copies of mtDNA [16]. Whilst a variety of variant callers exist, their ability to be tailored to the mitochondrial genome and account for the number of copies of mtDNA per cell may be unaccounted for [70]. MtDNA specific variant callers such as MToolBox, APOGEE and Mitoclass.1 have been developed and have enhanced ability to quantify heteroplasmies down to the 1% level [71–74]. Whilst valuable, it is important to question the relevance of detecting such minor variants when the threshold effect confers that 60–80% of cells must contain a variant before a phenotypic effect can be detected [16,17]. Nonetheless, it can be postulated that this increase in detail and complexity may provide greater specificity for defining associations between mtDNA and ADR, leading to a clearer route to translate findings to the clinic and drug safety setting.

The identification of mtDNA variant-specific, susceptible individuals to drug toxicity could prove invaluable in drug development and drug safety. However, given the high economic cost associated with large multicentre clinical trials, there is a need for new strategies to bridge preclinical and clinical outcomes [75]. Idiosyncratic drug-induced toxicities are difficult to detect preclinically during *in vivo* experiments due to the lack of genetic diversity amongst rodent populations. Additionally, the use of healthy rodents means that typical risk factors such as age and underlying disease are not considered during preclinical assessments [76]. Transmitochondrial cybrids may, therefore, offer an alternative preclinical screen in which the effects of mitochondrial genetic variation upon susceptibility to ADRs can be investigated, similar to the previously described adoption of their use in the investigations of cisplatin, aminoglycoside and linezolid [46,59,63]. Further support for this comes from a study of the mitochondrial poison rotenone, a complex I inhibitor, commonly used as a positive control for inducing mitochondrial dysfunction. Transmitochondrial cybrid investigations utilising osteosarcoma cybrids belonging to haplogroups, N1b, H1, J, T, U and K1, demonstrated that haplogroup J1-cybrids were associated with an increase in rotenone sensitivity, whereas cybrids belonging to haplogroup H1 and K1 were the most resistant [77]. The association between rotenone and mtDNA variation has also been observed when utilising mouse embryonic fibroblasts, with distinct mtDNA polymorphisms [76]. In addition to rotenone, there were also variations in response to the hepatotoxicants nefazodone, ketoconazole and tolcapone [76]. Overall, these studies suggest that the inclusion of alternative *in vitro* models, designed to be representative of increased mitochondrial genetic diversity, may improve preclinical screening for mitochondrial toxicants with an increased awareness of their risk of inducing ADRs in patients.

Overall, this review has highlighted that although mtDNA is an important factor in the onset of certain ADRs, current evidence suggests that in most cases, susceptibility cannot be explained by defining macro-haplogroup associations alone. For example, specific mtDNA mutations that may be determinants for some ADRs, can be found in multiple sub-haplogroups, in addition to the presence of haplogroup-specific nuclear DNA interactions or nuclear-modifier genes that alter the phenotype of the mtDNA variant [21,23,78]. However, the rapid advances in both the understanding of mitochondrial genetics and the technology with which to investigate it, coupled with a greater appreciation of its fundamental physiological and pathophysiological importance, present exciting opportunities to further establish mitochondrial genotype as an important topic in the study of pharmacogenomics and translational drug safety. In particular, efforts should be made to include mitochondrial NGS in ongoing genomic studies of ADRs which incorporate large cohorts of patients, for example, the study of antiretroviral agents. This would enable the importance of sub-haplogroup to be explored and also the opportunity for nuclear and mitochondrial genetic crossover to be explored alongside the effects of mtDNA mutational load. The mechanistic basis of any findings should be further investigated using *in vitro* experiments making use of defined transmitochondrial cybrid cells and primary cells in order to truly understand the molecular drivers of any mitochondrial associations which is vital to understand whether drug safety can be improved through an increased understanding of which individuals may be at increased risk or offer novel areas for mechanism-based safe drug-design or intervention therapy.

## Perspectives

- Current evidence supports that there is an important link between mtDNA variation and some forms of drug-induced ADRs. The identification of mtDNA variant-specific susceptible individuals to drug toxicity could prove invaluable in drug development and drug safety.
- Although clinical research has reported evidence to support a link between mitochondrial genotype and susceptibility to ADRs, their importance remains unclear due to limitations in study design, particularly the size and genotypic diversity.
- Rapid advances in the understanding of mitochondrial genetics, alongside the technology with which to investigate it, present exciting opportunities to further establish mitochondrial genotype as an important topic in the study of pharmacogenomics and translational drug safety.

## Open Access

Open access for this article was enabled by the participation of University of Liverpool in an all-inclusive *Read & Publish* pilot with Portland Press and the Biochemical Society under a transformative agreement with JISC.

## Abbreviations

ACTG: adults AIDS clinical trials group
ADP: adenosine diphosphate
ADRs: adverse drug reactions
AIDS: acquired immune deficiency syndrome
ART: antiretroviral therapy
ATP: adenosine triphosphate
BAX: BCL2-associated X protein
Ca^2+^: calcium
d4T: stavudine
ddI: didanosine
ETC: electron transport chain
FMD: flow-mediated dilation
HAART: highly active antiretroviral therapy
HIV: human immunodeficiency virus
mtDNA: mitochondrial DNA
NGS: next-generation sequencing
NHS: National Health Services
RPE: retinal epithelial
rRNA: ribosomal RNA
SNPs: single nucleotide polymorphisms
tRNA: transfer RNA
